# Saccharification of Lignocelluloses by Carbohydrate Active Enzymes of the White Rot Fungus *Dichomitus squalens*

**DOI:** 10.1371/journal.pone.0145166

**Published:** 2015-12-14

**Authors:** Johanna Rytioja, Kristiina Hildén, Susanna Mäkinen, Jari Vehmaanperä, Annele Hatakka, Miia R. Mäkelä

**Affiliations:** 1 Department of Food and Environmental Sciences, Division of Microbiology and Biotechnology, University of Helsinki, Helsinki, Finland; 2 Roal Oy, Rajamäki, Finland; INRA, FRANCE

## Abstract

White rot fungus *Dichomitus squalens* is an efficient lignocellulose degrading basidiomycete and a promising source for new plant cell wall polysaccharides depolymerizing enzymes. In this work, we focused on cellobiohydrolases (CBHs) of *D*. *squalens*. The native CBHI fraction of the fungus, consisting three isoenzymes, was purified and it maintained the activity for 60 min at 50°C, and was stable in acidic pH. Due to the lack of enzyme activity assay for detecting only CBHII activity, CBHII of *D*. *squalens* was produced recombinantly in an industrially important ascomycete host, *Trichoderma reesei*. CBH enzymes of *D*. *squalens* showed potential in hydrolysis of complex lignocellulose substrates sugar beet pulp and wheat bran, and microcrystalline cellulose, Avicel. Recombinant CBHII (rCel6A) of *D*. *squalens* hydrolysed all the studied plant biomasses. Compared to individual activities, synergistic effect between rCel6A and native CBHI fraction of *D*. *squalens* was significant in the hydrolysis of Avicel. Furthermore, the addition of laccase to the mixture of CBHI fraction and rCel6A significantly enhanced the amount of released reducing sugars from sugar beet pulp. Especially, synergy between individual enzymes is a crucial factor in the tailor-made enzyme mixtures needed for hydrolysis of different plant biomass feedstocks. Our data supports the importance of oxidoreductases in improved enzyme cocktails for lignocellulose saccharification.

## Introduction

Despite the enormous biotechnological potential, the number of biochemically characterized plant-polysaccharide-degrading enzymes from basidiomycete fungi is still scarce compared to the corresponding enzymes of ascomycetes. Basidiomycete white rot fungi are the only organisms in nature able to completely degrade lignocellulose including cellulose, hemicellulose and lignin polymers [1,2]. Cellulose, the most abundant biopolymer in nature, is built up of β-1,4-glucose units and hydrolysed by endoglucanases (EGs, E.C. 3.2.1.4), cellobiohydrolases (CBHIs, E.C. 3.2.1.176 and CBHIIs, E.C. 3.2.1.91) and β-glucosidases (BGLs, E.C. 3.2.1.21) into glucose monomers. The synergistic action of these glycoside hydrolases (GHs) has been well defined and they have been classified into the Carbohydrate-Active enZYme database (CAZy, http://www.cazy.org/, [3]) together with carbohydrate esterases, polysaccharide lyases, glycosyl transferases and auxiliary activities (AAs). In CAZy, CBHs are divided into two GH families, GH7 (CBHI, Cel7) and GH6 (CBHII, Cel6), and they hydrolyse cellulose from reducing and non-reducing end, respectively. White rot fungi are well known producers of lignin modifying oxidoreductases, namely laccases and class II peroxidases, but they also produce other oxidoreductases, such as cellobiose dehydrogenase (CDH, AA3_1, AA8; EC 1.1.99.18) and lytic polysaccharide monooxygenases (AA9), that were recently shown to play an important role in the degradation of carbohydrates [4].

The basidiomycete white rot fungus *Dichomitus squalens* efficiently degrades softwood and produces an extensive set of extracellular enzymes that degrade plant cell wall polysaccharides [5–7]. Few of its plant cell wall polymers modifying or degrading extracellular enzymes have been characterized at the protein level, namely two xylanases with CBHI activity [8], one α-arabinofuranosidase, two laccases and two manganese peroxidases (MnPs) [9–13]. However, none of these enzymes have been tested for enzymatic hydrolysis of plant biomass. The genome of *D*. *squalens* shows that the fungus possesses genes encoding a full range of classical hydrolytic cellulases including one *cel6* and three *cel7*, five EG encoding *cel5* and eight BGL encoding *cel3* genes, as well as one *cdh* and 15 *aa9* genes encoding carbohydrate-oxidizing enzymes [14,15].

Novel cellulolytic enzymes and their characteristics have been studied for past years to overcome economic and technological bottlenecks of lignocellulose utilization in second generation biofuel and biorefining applications. One of the main obstacles in the enzymatic saccharification of plant biomass is efficient breakdown of crystalline cellulose into glucose [16]. Prior to the enzymatic saccharification, pretreatment of the plant biomass is needed to loosen the lignocellulose structure, e.g. by thermochemical steam explosion. Pretreatment also removes part of hemicelluloses and removes or modifies lignin, thus introducing more free cellulose chains for enzymatic hydrolysis [16]. However, the modified lignin can hinder the activity of cellulases by binding to them nonspecifically [17,18]. Oxidative laccase enzymes (AA1_1, EC 1.10.3.2) catalyse one-electron oxidation of a variety of phenolic and low-redox potential compounds [2], and have been reported to enhance pretreatment of plant biomass and improve enzymatic hydrolysis [19,20]. This has been suggested to be due to loosening the lignocellulose structure and detoxification of plant-biomass derived phenolic inhibitor compounds, respectively. Basidiomycete enzymes represent a so far unused source that can provide more efficient catalysts for the enzyme palette for plant biomass saccharification [2].

In the present work, we focused on functional characterization of CBH enzymes of *D*. *squalens* and their ability to saccharify lignocellulosic substrates. CBHIs were produced as native enzyme pool, whereas CBHII (rCel6A) enzyme of *D*. *squalens* was produced recombinantly due to lack of enzyme activity assay for detecting only CBHII activity. *Trichoderma reesei* was used as the expression host, as it is commonly applied in industrial production of cellulolytic enzymes. In addition, the native CBHI fraction and CDH were purified and characterized from the submerged microcrystalline cellulose (Avicel) liquid cultures of *D*. *squalens*. Enzymes were shown to hydrolyse sugar beet pulp (SBP), wheat bran (WB) and Avicel. The results also confirmed synergism between CBH enzymes especially in the saccharification of Avicel. The addition of laccase further enhanced the sugar yield from SBP and Avicel, thus suggesting the importance of both hydrolytic and oxidative activities in saccharification of lignocellulosic feedstocks.

## Materials and Methods

### Fungal cultivations

*Dichomitus squalens* FBCC312 (A-670) was obtained from the Fungal Biotechnology Culture Collection (FBCC, email: fbcc@helsinki.fi), Department of Food and Environmental Sciences, University of Helsinki, Finland. Fungus was maintained on 2% malt agar plates [2% (w/v) malt extract (Biokar, France), 2% (w/v) agar agar (Biokar, France)]. For inoculum, the fungus was cultivated in 75 ml liquid 2% (w/v) malt extract medium in 250 ml Erlenmeyer flasks for 8 days at 28°C, and homogenised in Waring blender. The homogenate (3–4 ml) was used to inoculate 100 ml of 1% Avicel medium, pH 6.0, which contained 2.5 g/l meat peptone (Lab M Limited, UK), 1 g/l yeast extract (Lab M Limited, UK), 1 g/l potassium dihydrogen phosphate (Sigma-Aldrich, Japan), 0.5 g/l magnesium sulphate (Merck, Germany) and 1% (w/v) Avicel® PH-101 cellulose (Fluka, Ireland) with or without 0.25% (w/v) Tween20 (Sigma Aldrich, Germany). The agitated (120 rpm) cultivations were performed in 250 ml baffled flasks and incubated for 29 days at 28°C. The cultivations were sampled three times a week for extracellular enzyme activity measurements. For enzyme purification, the culture liquids were collected after 6 to 10 days of cultivation and frozen at -20°C prior to purification.

### Enzyme activity measurements

Extracellular enzyme activities were determined from three replicate cultures. All the measurements were performed in 96-well plates as triplicates using Tecan Infinite M200 plate reader (Tecan, Austria), except CDH activity, which was measured by using Shimadzu PharmaSpec UV-Vis-1700 spectrophotometer. The activities are expressed as nkat/ml (10^−9^ mol/s/ml). Cellulase (CBH, EG and BGL) and xylanase activities were measured in 50 mM Na-citrate buffer, pH 5.0, at 45°C, as previously described [7]. CBH activity was determined with 1.6 mM 4-methylumbelliferyl-β-D-lactoside (MULac, Biokemis, Russia) as a substrate [21]. EG and xylanase activities were measured using 1% (w/v) hydroxyethyl cellulose (Sigma, USA) [22] and 1% (w/v) birch or beech xylan (Sigma, Germany) [23] as substrates, respectively. Reducing sugars were measured by using dinitrosalisylic acid (DNS) method at 540 nm [24]. Activity for BGL was determined with 0.9 mM 4-nitrophenyl β-D-glucopyranoside (Applied Chemical Laboratories, USA) as a substrate.

CDH activity was assayed with 30 mM cellobiose as a substrate and 300 μM 2,6-dichlorophenol-indophenol (Fluka, Austria) as an electron acceptor by following the decrease of absorbance at 520 nm [25] in 100 mM Na-acetate buffer, pH 4.5, at 45°C. Laccase activity was inhibited with 4 mM sodium fluoride (Riedel-de Haën, Germany).

Laccase activity was monitored at 476 nm by detecting the oxidation of 2,6-dimethoxyphenol (Aldrich, Germany) in 50 mM Na-malonate buffer, pH 3.0, at 25°C [26]. Manganese peroxidase (MnP) activity was determined by following the formation of Mn^3+^-malonate complex at 270 nm in 50 mM Na-malonate buffer, pH 4.5, at 25°C [27].

### Amplification and cloning of *D*. *squalens cel6a* gene

Total RNA was extracted from fungal mycelium obtained from the 1% Avicel cultures after 7 days of incubation. The mycelium was homogenised in Lysing Matrix C tubes (M.P. Biomedicals, USA) by using FastPrep® apparatus (M.P. Biomedicals, USA) and RNA was extracted with TRI Reagent (Sigma-Aldrich, USA) according to the instructions of the manufacturer. RNA was treated with RNase-free DNase (Fermentas, Lithuania) and cDNA was synthesised with Smart RACE cDNA Amplification Kit (Clontech, USA) according to the instructions of the manufacturer. The 30 μl reactions contained 1 μg RNA, 400 U Superscript III reverse transcriptase (Invitrogen, USA), 4 μl of 5x first strand buffer, 0.6 μM 3´-RACE primer, 0.6 μM SMART II oligonucleotide, 13 mM dithiothreitol (Fermentas, Lithuania) and 1.3 mM dNTPs (Fermentas, Lithuania).

The full-length ORF of *cel6a* (GenBank: KT595235) was amplified from the cDNA with gene specific primers (sense: ATGTCCAAGTTTGCGACACTCTG, antisense: TTACAGCGGGGGGTTCGCCTGGGA) designed according to the transcript model #152348 from the whole genome sequence of *D*. *squalens* LYAD-421 SS1 (http://genome.jgi-psf.org/Dicsq1/Dicsq1.home.html). PCR reaction in 1x Dynazyme buffer (Finnzymes, Finland) contained 0.5 μl cDNA template, 0.3 mM dNTPs (Fermentas. Lithuania), 0.4 μM 5´and 3´ primers and 0.5 μl DynazymeII DNA polymerase (Finnzymes, Finland). PCR was performed with initial denaturation at 95°C for 5 min, following with 40 cycles of denaturation at 94°C for 35 s, annealing at 55°C for 1 min then increasing the temperature to 72°C at 0.2°C/s, elongation at 72°C for 2 min, and final extension at 72°C for 10 min. The amplification product was run on 1% agarose gel, purified with GeneJET Gel Extraction kit (Fermentas, Lithuania) according to the instructions of the manufacturer, cloned into the pJET1.2/blunt vector (Fermentas, Lithuania) and sequenced (Macrogen Corp., The Netherlands).

### Production of *D*. *squalens* Cel6A in *Trichoderma reesei*

The *D*. *squalens cel6a* gene sequence was codon optimised for production in *Trichoderma reesei* and the synthetic gene (GenScript, USA) with the native *D*. *squalens cel6a* signal sequence was exactly fused to the *T*. *reesei cbh1 (cel7A)* promoter. A *Bam*HI site was created at the 3´-end of the gene after the stop codon, to enable ligation to the *T*. *reesei cbh1* terminator in the expression vector. The *amdS* marker gene was ligated 3´ of the *cbh1* terminator. The construction of the vector for the expression of the *D*. *squalens cel6a* gene was analogous to that described previously for a heterologous xylanase [28]. A 6.7 kb linear expression cassette isolated from the *Escherichia coli* vector after *Not*I digestion was used for transforming the *T*. *reesei* protoplasts. The *T*. *reesei* strain used as a host for transformation (a proprietary strain of Roal Oy) does not produce any of the four major *T*. *reesei* cellulases (CBHI, CBHII, EGI, EGII). The transformation was performed as described previously [29,30]. The transformants were purified on selection plates through single conidia before being sporulated on potato dextrose (PD) agar (Difco, France) slants. The transformants were inoculated from the PD slants to shake flasks containing 50 ml of complex lactose-based cellulase inducing medium [31] buffered with 5% KH_2_PO_4_ at pH 5.5. The production of the rCel6A protein was analysed from the transformants after growing them for 7 days at 30°C, 250 rpm, by sodium dodecyl sulphate-polyacrylamide gel electrophoresis (SDS-PAGE) in 12% Criterion™ XT Bis-Tris gels (Bio-Rad, USA). The best enzyme producing *T*. *reesei* transformant in the shake flask cultivations was selected to be cultivated in a laboratory scale bioreactor in a cellulase inducing complex medium. The spent culture medium obtained from the cultivation was used without further purification for the enzymatic hydrolysis experiments. Inquiries concerning the availability of the *T*. *reesei* strain and plasmids can be forwarded to Roal Oy, Rajamäki, Finland.

### Purification and characterization of native *D*. *squalens* CBHI and CDH enzymes

The frozen culture liquid was melted, filtered through Miracloth (Calbiochem, USA) and Whatman GF/C glass microfibre filters (England) and concentrated by using Filtron Minisette apparatus (with 10.000 NMWL filter casettes) and Amicon ultrafiltration unit with 10.000 NMWL polyethersulphone membranes (Sartorius, Germany) at 4°C. CBHI and CDH activities were fractionated chromatographically with Äkta Explorer apparatus (GE Healthcare, Sweden). After each chromatographic step, the protein fractions containing CBHI and CDH activity were pooled and concentrated with 10 kDa cut-off ultra-concentration tubes (Corning, UK). First, three steps of anion exchange chromatography were performed. The samples were fed into Q Sepharose FF and HiTrap Q FF columns (GE Healthcare, Sweden) in 10 mM triethylamine (TEA, Sigma Aldrich, Germany) buffer (pH 7.5) and the proteins were eluted with linear or stepwise NaCl gradient from 0 to 1 M in 20 mM TEA buffer, respectively. The pooled fractions were further purified in MonoQ GL 5/5 column (GE Healthcare, Sweden) using 10 mM Na-acetate buffer (pH 6.5) and eluted with a stepwise NaCl gradient from 0 to 1 M. As the last chromatographic step, the proteins were separated with size exclusion chromatography in Superdex 200 100/300 GL column (GE Healthcare, Sweden) with 0.15 M NaCl in 50 mM phosphate buffer (pH 7.0).

Chromatographically purified protein fractions were separated by SDS-PAGE in MiniProtean TGX gels (Bio-Rad, USA) and analytical isoelectric focusing (IEF) with Multiphor II apparatus (Pharmacia, Sweden) in 7.5% polyacrylamide gels. The pH gradient (from 3.5 to 8.2) of the IEF gels was obtained with a mixture of ampholytes (Pharmalyte^TM^ 2.5–5 and 3.5–10, GE Healthcare, Sweden) and was measured using a surface pH electrode (Orion^TM^ ROSS^TM^ 8135SCU, USA). The proteins in SDS-PAGE and IEF gels were visualised with EZBlue staining reagent (Sigma-Aldrich, USA). Protein concentration of the purified fractions was determined by using bicinchoninic acid method (Pierce BCA Protein Assay kit, Thermo Scientific, USA). The internal peptides of the purified CBHI fraction and CDH were obtained from LC-MS/MS sequencing at the Proteomics Unit, Institute of Biotechnology, University of Helsinki, Finland.

Temperature and pH optima of the purified CBHI fraction were determined at temperatures ranging from 45°C to 70°C, and with a pH range from 3.0 to 7.0, respectively, using MULac as a substrate. Thermostability was examined by incubating the enzyme in 50 mM Na-citrate buffer, pH 5.0, at temperatures from 30°C to 70°C for 1 to 60 min, after which the residual activity was measured at 45°C. The pH stability of the CBHI fraction was determined by incubating the enzyme in 50 mM Na-citrate buffer at pH from 2.5 to 6.0 for 1 to 60 min, after which the residual activity was measured at pH 5.0.

### Enzymatic hydrolysis

Enzymatic hydrolysis of Avicel, sugar beet pulp (powdered, SBP; Danisco Ingredients, Denmark) and wheat bran (powdered, WB; Danisco Ingredients, Denmark) was conducted in 250 μl reactions with 1% (w/v) solid concentration in 50 mM Na-citrate buffer, pH 4.0, for 4 h at 50°C under agitation (1400 rpm). The hydrolysis reactions contained either individual enzymes or enzyme combinations in the following amounts per mg of substrate: 5 μg of the purified fraction of *D*. *squalens* CBHI, 5 or 10 μg of *D*. *squalens* rCel6A, 0.5 and 1 μg of purified *D*. *squalens* CDH and 0.5 and 1 μg of a commercial laccase from *Myceliophthora thermophila* (Novozym 51003, Novozymes, Denmark). All the experiments were carried out in triplicates. After incubation, the reactions were placed on ice and centrifuged at 2250 × g (Eppendorf 5804R) for 10 min at 4°C. The supernatants were collected and the amount of reducing sugars was determined by using DNS method. The amount of released reducing sugars was calculated as % of the theoretical carbohydrate content of Avicel (94%, [32]), SBP (55%, [33]) and WB (60%, [34]). The spent culture medium of a *T*. *reesei* host strain lacking the four major cellulases and without the *cel6a* expression cassette (a proprietary strain of Roal Oy) was used as a background control and the amount of reducing sugars released by it was subtracted from the results of rCel6A. In addition, the background control and the purified fraction of *D*. *squalens* CBHI were used in the hydrolysis reactions to confirm that there was no synergistic effect between *T*. *reesei* side activities and *D*. *squalens* CBHI fraction. The control medium was loaded based on the same level of the xylanase activity than the rCel6A preparation had.

### Statistical analyses

Differences in the amounts of the released reducing sugars were estimated with ONE-way ANOVA-test for the data that was normally distributed according to the Kolmogorov-Smirnov test. The p-values <0.05 were considered to be significant. Analyses were calculated with PASW Statistics 18 software (IBM, USA).

## Results and Discussion

### Production of carbohydrate acting enzymes by *D*. *squalens*

To characterize the carbohydrate active enzymes of the white rot fungus *D*. *squalens*, the fungus was cultivated in 1% Avicel medium, from which a variety of carbohydrate depolymerizing enzyme activities were detected in our previous study [7]. In this study, similar profile of extracellular EG, BGL, xylanase and laccase activities was observed (S1 Fig) as previously [7]. In addition, a minor MnP activity (from 0.1 to 0.2 nkat/ml) was detected from the Avicel cultures at days 12 and 22 (S1 Fig).

CDH activity was not detected from the 1% Avicel medium. Therefore 0.25% of the surfactant Tween20 was amended to the medium to enhance secretion of enzymes into the growth liquid [35]. As a result, CDH activity was observed (S1 Fig). The activity appeared after 10 days of growth, peaking up to 5 nkat/ml on day 24, and accumulated until the end of the cultivation. In addition to the possible increase of permeability of cell membrane [35], polyoxyethylene sorbate compounds (Tweens) have also been reported to positively influence mycelial morphology resulting with increased fungal biomass in shake flask cultivations [36]. Tweens may also protect extracellular enzymes from mechanical inactivation caused by agitation [37]. However, the accumulation of EG and CBHI activities was delayed in Tween20-amended cultures compared to the cultures without the surfactant (S1 Fig).

### Characterization of CBHI and CDH of *D*. *squalens*

**Fig 1:**
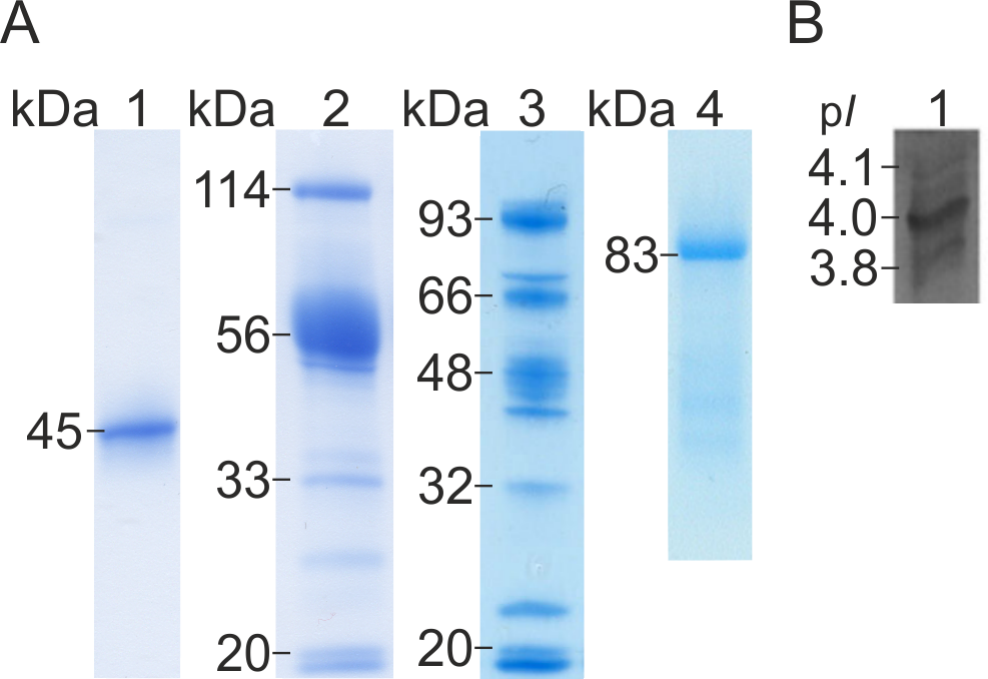
Gel electrophoretic (SDS-PAGE) separation and isoelectric focusing (IEF) of *D*. *squalens* enzymes. (A) SDS-PAGE of chromatographically purified CBHI fraction, heterologously produced rCel6A, and purified CDH of *D*. *squalens*. Lane 1, CBHI fraction; lane 2, rCel6A; lane 3, *T*. *reesei* culture filtrate without *cel6a* insert; lane 4 CDH. (B) IEF analysis of CBHI fraction of *D*. *squalens*.

In order to characterize the CBH enzymes of *D*. *squalens*, the native CBHI pool was chromatographically purified from 1% Avicel cultures. When the CBHI fraction was separated by gel electrophoresis (SDS-PAGE), one protein band with molecular mass of 45 kDa was detected (Fig 1A). This is slightly lower than the theoretical molecular masses of the three translated CBHI-encoding genes of *D*. *squalens* (protein IDs #96488, #100956 and #131122, http://genome.jgi-psf.org/Dicsq1/Dicsq1.home.html) varying from 48.8 kDa to 49.0 kDa (http://web.expasy.org/compute_pi/). However, analytical IEF revealed several isoforms of CBHs with p*I* values from 3.8 to 4.1 (Fig 1B), suggesting that *D*. *squalens* produced the three different CBHI isoenzymes simultaneously in the Avicel cultures. This is in line with the theoretical p*I* values of *D*. *squalens* CBHI proteins ranging from 4.24 to 4.93 (http://web.expasy.org/compute_pi). Rouau and Odier purified two enzymes (Ex1 and Ex2) from another strain of *D*. *squalens*, CBS432-34, showing both CBHI and xylanase activities [8]. In contrast to Ex1 and Ex2, the CBHI fraction in our work did not show any activity towards xylan, thus confirming that the CBHI fraction is different from Ex1 and Ex2 enzymes. The molecular masses of Ex1 and Ex2 (39.0 and 36.0 kDa, respectively) were also lower and the isoelectric points (4.6 and 4.5, respectively) were slightly higher than those of the CBHI fraction characterized in this study.

Three internal peptide sequences obtained with LC-MS/MS analysis from the CBHI fraction (SVVLDSNWR, YGTGYCDTQCPHDIK, LYVQNGKVIANSK) were found to be identical with the translated CBHI-encoding genes *cel7a* and *cel7b* of *D*. *squalens* FBCC312 [7,14]. Furthermore, two peptides (MGDQTFLGPGK, DGCDFNSWR) were identical with the translated CBHI-encoding gene *cel7a* and two peptides (LYVQNGKVIANSK, DGCDFNSWR) with the translated CBHI-encoding gene *cel7c* of *D*. *squalens* FBCC312 [14]. These results strongly suggest that the chromatographically purified CBHI fraction was a mixture of the three CBHI proteins encoded by the genome of *D*. *squalens* that were produced simultaneously in the cellulose cultures. This is in line with the concurrent expression of CBHI-encoding genes that we have observed in the Avicel cultures of *D*. *squalens* [7]. Typically, genomes of white rot fungi harbour several CBHI isoenzymes encoding genes [1] and their simultaneous production has been detected in the secretome studies of *Bjerkandera adusta*, *Ganoderma* spp., *Phanerochaete chrysosporium* and *Phlebia brevispora* in aspen wood cultures [38,39].

The purified *D*. *squalens* CDH was detected as a single protein band with the molecular mass of 83 kDa (Fig 1A), which corresponds with the previously characterized white rot fungal CDH proteins with molecular masses ranging from 81 to 113 kDa [1]. The LC-MS/MS analysis of the purified CDH resulted with three internal peptides (KVLLLER, VILSAGSFGTPR and SGVFAGASPK) that were identical with the translated *cdh* gene of *D*. *squalens* FBCC312 [7]. We have previously shown the constitutive expression of the *cdh* during the growth of *D*. *squalens* in Avicel medium [7]. Our result is also in line with the study demonstrating that cellulose (α-cellulose or microcrystalline cellulose, Avicel) is a good substrate for CDH production in the plant pathogenic basidiomycete *Sclerotium* (*Athelia*) *rolfsii* [40]. These results support the confidence that CDH has a role in cellulose depolymerization.

**Fig 2:**
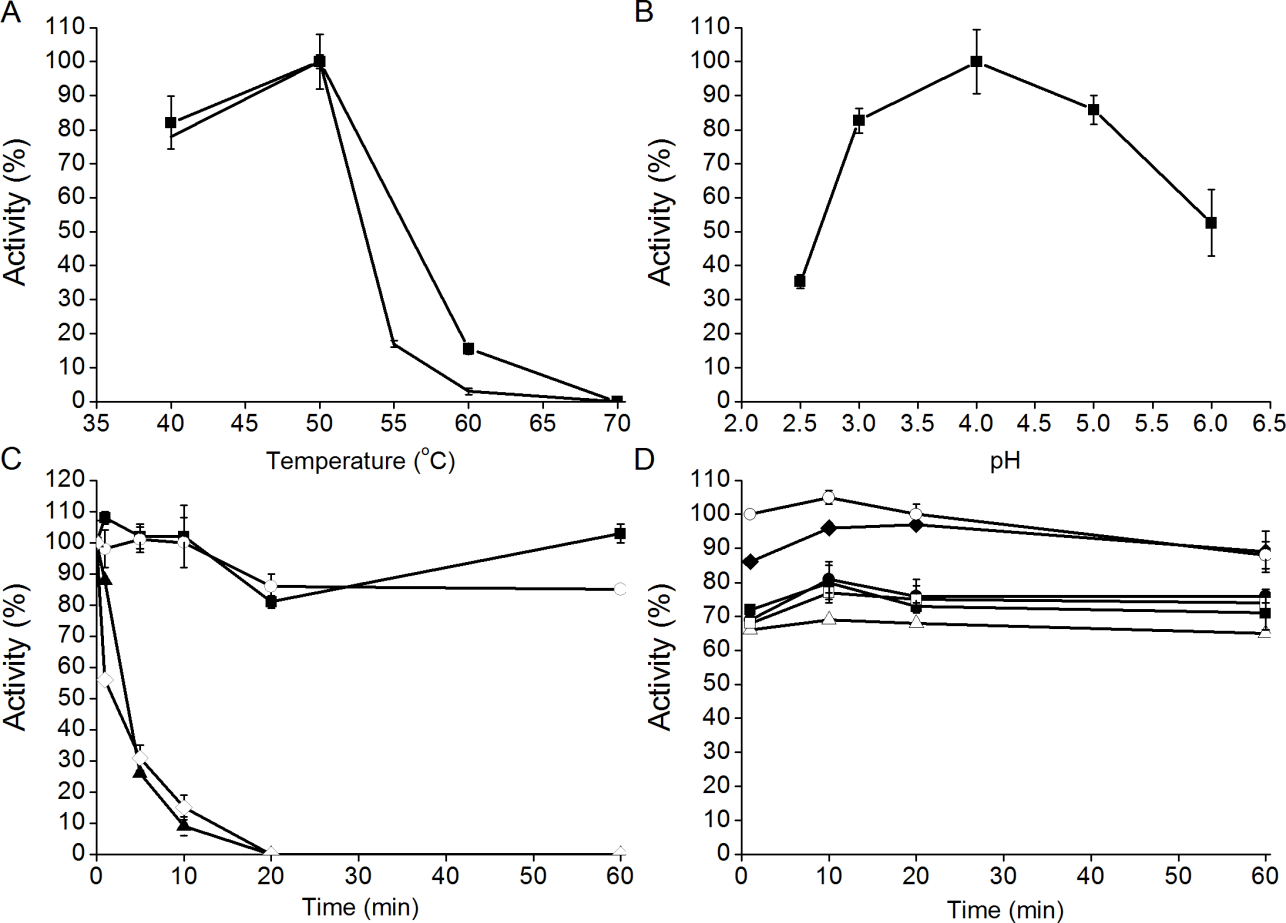
Characterization of CBHI fraction of *D*. *squalens* **(**A) Temperature (10 min reaction, ■; 1 h reaction, ━) and (B) pH optimum, (C) thermostability at 40 (■), 50 (○), 60 (▲) and 70°C (◇), and (D) pH stability at pH 3.0 (■), pH 4.0 (●), pH 5.0 (△), pH 6.0 (◆), pH 6.5 (□) and in distilled water (○) of the purified CBHI fraction. Reactions were conducted in 50 mM sodium citrate buffer at pH 5.0 with MULac as substrate, except pH stability, which was studied in various buffer pH values and in distilled water. Standard deviations of the activities of three technical replicates are shown as error bars.

The temperature and pH optima of the purified CBHI fraction of *D*. *squalens* were 50°C and 4.0, respectively, when MULac was used as a substrate (Fig 2A and 2B). The CBHI maintained its activity for 60 min at 50°C (Fig 2C), and was stable in 50 mM Na-citrate buffer from pH 3.0 to pH 6.0 (Fig 2D). The pH and temperature ranges of *D*. *squalens* CBHIs correspond with the previously characterized white rot fungal CBHIs [1]. The analysis also confirmed that the CBHI fraction was different from the previously characterized Ex1 and Ex2 enzymes of *D*. *squalens* with temperature optima of 60°C [8]. *D*. *squalens* CBHI fraction was less thermotolerant than Cel7A from *T*. *reesei*, which reaches its highest activity at 60°C [41]. The pH optimum of *D*. *squalens* CBHI fraction (pH 4.0) was more acidic than that of *T*. *reesei* Cel7A (pH 5.0) [41].

### Production of recombinant CBHII of *D*. *squalens* in *T*. *reesei*

In this work, the CBHII (Cel6A) enzyme of the white rot fungus *D*. *squalens* was successfully produced in *T*. *reesei*. To our knowledge, this is the first report of heterologous production of basidiomycete CBHII enzyme in the industrially important *T*. *reesei* host. Previously, basidiomycete CBHIIs from *Irpex lacteus* and *Coprinopsis cinerea* have been produced in *Pichia pastoris* and *Escherichia coli*, respectively [42–44]. A CBHII encoding fragment, corresponding to *cel6a* gene model of *D*. *squalens*, was amplified from cDNA originating from mycelium grown in Avicel cultures. The *cel6a* gene sequence was codon optimized and the synthetic sequence was transformed into *T*. *reesei*. The molecular mass of the recombinant protein was 56 kDa (Fig 1A), which was slightly higher than the theoretical molecular mass, 45.3 kDa, of the mature *D*. *squalens* Cel6A (ExPASy pI/Mw tool; http://web.expasy.org/compute_pi/), most probably due to glycosylation of the protein. From the culture liquid of *T*. *reesei* control strain (*T*. *reesei* host strain lacking the four major cellulases and without the *cel6a* expression cassette) no major protein bands with molecular mass of 50–60 kDa were detected (Fig 1A). Typically molecular masses of basidiomycete CBHIIs vary between 50 and 60 kDa [1]. No CBHI activity was detected with MULac as a substrate. Nevertheless, *T*. *reesei* rCel6A culture filtrate contained 52.7 nkat/mg of xylanase, 4.1 nkat/mg of EG and 3.0 nkat/mg of BGL as side activities, and therefore the culture liquid of the *T*. *reesei* control strain was used as a background control in hydrolysis experiments.

### Enzymatic hydrolysis

**Fig 3:**
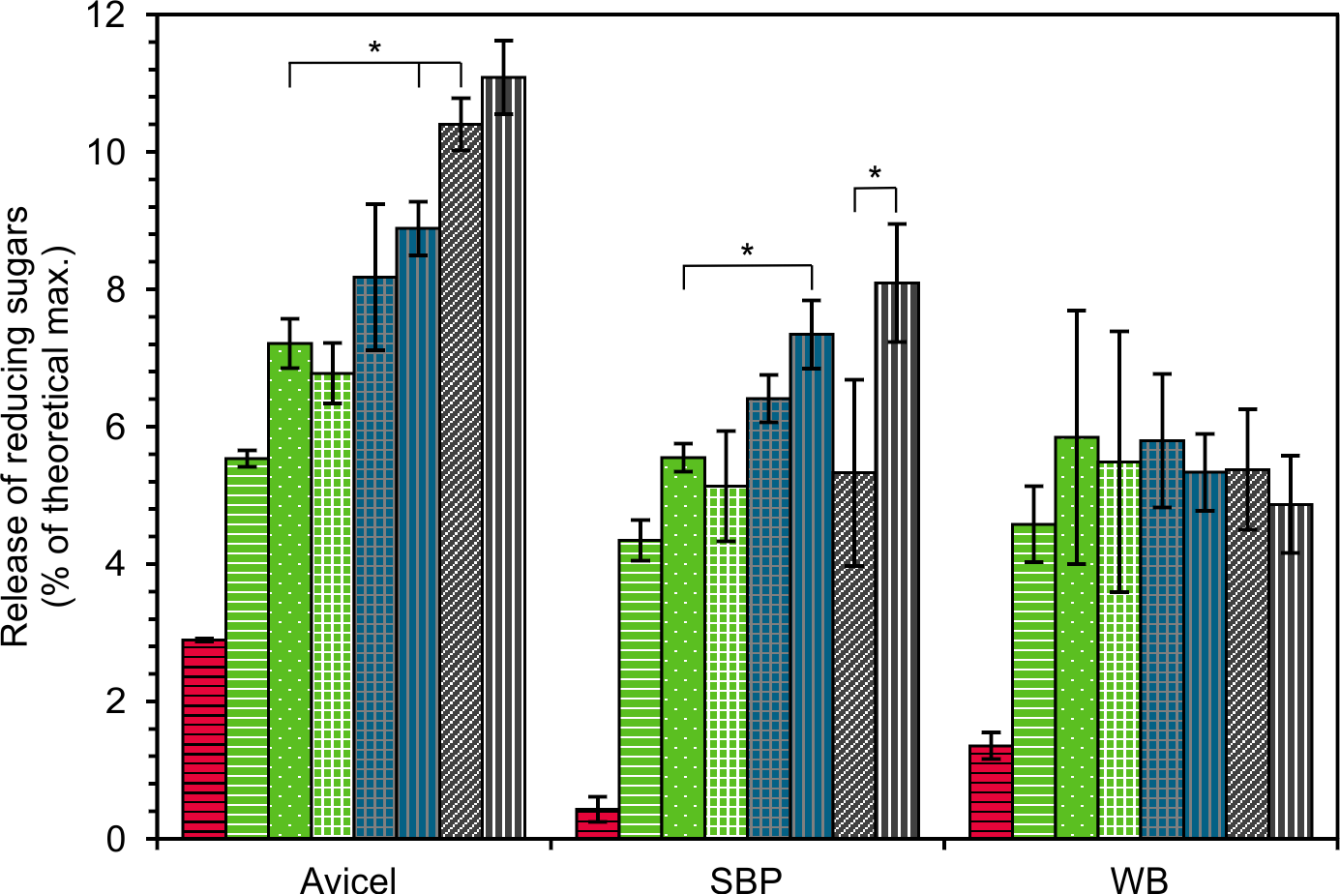
Hydrolysis of 1% (w/v) Avicel, sugar beet pulp (SBP) and wheat bran (WB). Reactions with CBHI fraction (5 μg/mg, red with horizontal stripes); rCel6A (5 μg/mg green with white horizontal stripes; 10 μg/mg, green with white dots); rCel6A and CDH (10 μg/mg and 1 μg/mg, respectively, green with white grid); rCel6A, CDH and laccase (10, 0.5 and 0.5 μg/mg, respectively, blue with grey grid); rCel6A and laccase (10 and 1 μg/mg, respectively, blue with grey vertical stripes); CBHI fraction and rCel6A (5 and 5 μg/mg, respectively, grey with diagonal stripes) or CBHI fraction, rCel6A and laccase (5, 5 and 1 μg/mg, respectively, grey with white vertical stripes), for 4 h at 50°C. Significant differences (p<0.05) in the amount of released reducing sugars between the compared reactions are indicated by asterisk.

As there is no specific measurement method for CBHII activity, the function of *D*. *squalens* rCel6A was verified by saccharification assays. In addition to *D*. *squalens* rCel6A, CBHI, CDH, and *M*. *thermophila* laccase enzyme preparations were tested for the hydrolysis of crude plant biomass substrates SBP and WB, and microcrystalline cellulose Avicel. Both rCel6A and CBHI fraction were able to hydrolyse all the substrates (Fig 3).

With CBHI (5 μg/mg), hydrolysis yield from Avicel, SBP and WB was low (2.9%, 0.4% and 1.4% of the theoretical maximum, respectively). However, this is comparable with *T*. *reesei* Cel7A for which the hydrolysis yield from Avicel has been 2.5–4% when the reactions have been conducted at 45–55°C [45]. The rCel6A, which contains a cellulose binding domain (CBM1), was more efficient in releasing reducing sugars from all of the substrates than CBHI fraction which lacks the CBMs. Similarly, ascomycetous CBHs (GH7 and GH6) with CBMs have been shown to be more efficient in hydrolysis at low substrate concentration (1% w/w) than CBHs that lack CBMs [46].

The synergistic effect of CBHs (CBHI and rCel6A) was significant in the hydrolysis of Avicel. After addition of 5 μg of both rCel6A and CBHI fraction per mg of Avicel, the amount of released sugars was 1.2-fold higher than the calculated sum of released sugars from the hydrolysis of the individual CBHs (Fig 3). Similar synergistic effect has also been reported for basidiomycetes *I*. *lacteus* and *P*. *chrysosporium* CBHs with Avicel [42,47]. When similar enzyme loading was used with complex substrates, SBP and WB, the synergistic effect of CBHs was not significant or detected, respectively. In comparison to Avicel, lower sugar yields were achieved from hydrolysis of SBP and WB with both *D*. *squalens* CBHs (Fig 3) due to the complexity of the lignocellulosic feedstocks.

*P*. *chrysosporium* CDH has been shown to decrease the inhibitory effect of cellobiose on CBHI reaction by oxidation of cellobiose to cellobionolactone and thus improve the CBHI activity [48]. CDH has also been hypothesized to be a link between the cellulolytic and ligninolytic reactions [49] and recently a very strong synergy was detected for CDH and LPMO [50]. In this study, addition of *D*. *squalens* CDH solely or with laccase to the hydrolysis reactions of rCel6A (10 μg/mg) did not alter the amount of released sugars from any of the substrates (Fig 3).

Addition of *M*. *thermophila* laccase to the mixture of CBHI fraction and rCel6A resulted in significantly improved saccharification of SBP by 1.5-fold in comparison to the sugar yield obtained in the reaction without laccase. The important role of laccase was also shown by 1.3-fold increase of SBP hydrolysis when rCel6A and laccase were used together (Fig 3). However, no statistically significant effect of laccase was detected with WB. Laccases have been applied both in pretreatment and hydrolysis of plant biomass where they have been suggested to act by loosening the lignocellulose structure and by detoxifying the phenolic inhibitory compounds [51]. Laccase treatment has been shown to change the binding properties of cellulases onto lignin [20]. When steam-pretreated spruce was treated with laccase, the amount of bound cellulases was lower, whereas in the case of steam-pretreated giant reed (*Arundo donax*) laccase treatment caused increased binding of cellulases to lignin [20].

SBP is a major by-product of the sugar refining industry and it contains 1–2% (as dry weight) lignin [52] and is rich in glucose, arabinose and uronic acids [53]. Compared to SBP, WB is rich in lignin. WB contains approximately 10% (as dry weight) lignin [54], and majority of its carbohydrate content is glucose, xylose and arabinose [53]. Our results suggest that the lignin in SBP was altered by laccase resulting with easier access of CBHs to cellulose fibres. Interestingly, laccase addition to rCel6A also improved the sugar yield from Avicel where lignin content is very low (<1% of dry weight) [55]. In this work laccase had no effect on saccharification of WB most probably due to the high lignin content of WB. One explanation may be that the commercial laccase used in this study originated from *M*. *thermophila* which is an ascomycete species and possibly not involved in lignin modification in nature. However, promising results in the hydrolysis of lignin-rich plant biomass have been achieved with basidiomycete laccases (reviewed in [51]). Therefore, a more detailed understanding of the modifications to biomass caused by laccases from different fungal sources is needed in order to improve the yield of enzymatic saccharification.

## Supporting Information

S1 Fig Extracellular enzyme activities detected during the cultivation of *D*. *squalens*. Cultivation in (A) 1% (w/v) Avicel medium and in (B) 1% (w/v) Avicel medium supplemented with 0.25% (w/v) Tween20. CBHI (■), EG (●), BGL (▲), xylanase (□), laccase (○), manganese peroxidase (△), cellobiose dehydrogenase (◆). Standard deviations of the activities of three biological replicates are shown as error bars.
(PDF)
