# Supplementary figures and images for: Saccharification of Lignocelluloses by Carbohydrate Active Enzymes of the White Rot Fungus Dichomitus squalens
Source: PLoS One. 2015 Dec 14;10(12):e0145166. doi: 10.1371/journal.pone.0145166 (PMC4682842; doi:10.1371/journal.pone.0145166)

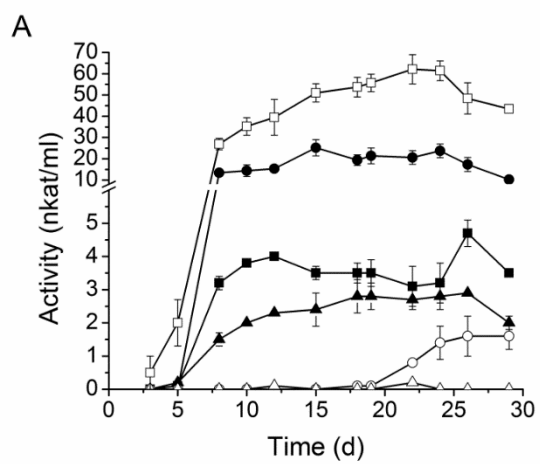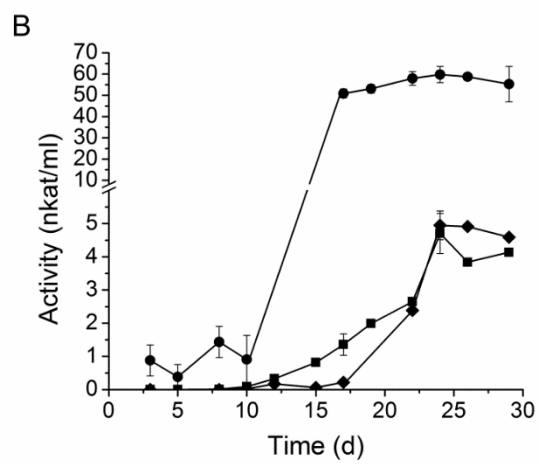

Supplement: S1 Fig — Cultivation in (A) 1% (w/v) Avicel medium and in (B) 1% (w/v) Avicel medium supplemented with 0.25% (w/v) Tween20. CBHI (■), EG (●), BGL (▲), xylanase (□), laccase (○), manganese peroxidase (△), cellobiose dehydrogenase (◆). Standard deviations of the activities of three biological replicates are shown as error bars. (PDF) [file pone.0145166.s001.pdf]
